# Analysis of IGHA1 and other salivary proteins post half marathon in female participants

**DOI:** 10.7717/peerj.15075

**Published:** 2023-05-11

**Authors:** Yosuke Maruyama, Tomoaki Seki, Seiichi Ando, Hiroki Tanabe, Hitoshi Mori

**Affiliations:** 1 Faculty of Health and Welfare Science Department of Nutritional Sciences, Nayoro City University, Nayoro, Hokkaido, Japan; 2 National Institute of Fitness and Sports Kanoya, Kanoya, Kagoshima, Japan; 3 Clinical Nutrition and Internal Medicine, Kobe Women’s University, Kobe, Hyogo, Japan; 4 Graduate School of Bioagricultural Sciences, Nagoya University, Nagoya, Aichi, Japan

**Keywords:** Saliva, Sports, Proteome, IGHA1

## Abstract

**Background:**

High-intensity exercise (HIE), such as that in marathons and triathlons, suppresses transient local and systemic immunity. Serum and salivary immunoglobulin heavy constant alpha 1 (IGHA1) are major markers of immunosuppression by HIE. Although much is known about the systemic immunosuppressive response, little is known about its local response in the oral cavity, lungs, bronchial tubes, and skin. The oral cavity allows bacteria or viruses to enter the body. Saliva covers the epidermis of the oral cavity and plays an important role in the local stress response by preventing infection. In this study, we examined the properties of saliva secreted during the local stress response for half-marathon (HM) induced IGHA1 protein expression using quantitative proteomics.

**Methods:**

The Exercise Group (ExG) (19 healthy female university students) participated in a HM race. The Non-Exercise Group (NExG) (16 healthy female university students) did not participate in the ExG. The ExG saliva samples were collected 1 h pre and 2 h and 4 h post-HM. The NExG saliva samples were collected at the same time intervals. The saliva volume, protein concentration, and relative IGHA1 expression were analyzed. In addition, 1 h pre and 2 h post- HM saliva samples were analyzed by iTRAQ. The identified factors in iTRAQ were analyzed for the ExG and the NExG using western blotting.

**Results:**

We identified kallikrein 1 (KLK1), immunoglobulin kappa chain (IgK), and cystatin S (CST4) as suppression factors, as well as IGHA1, which has been reported to be an immunological stress marker. IGHA1 (*p* = 0.003), KLK1 (*p* = 0.011), IGK (*p* = 0.002), and CST4 (*p* = 0.003) were suppressed 2 h post-HM compared with their levels pre HM, and IGHA1 (*p* < 0.001), KLK1 (*p* = 0.004), and CST4 (*p* = 0.006) were suppressed 4 h post-HM. There was also a positive correlation between IGHA1, IGK, and CST4 levels at 2 and 4 h post-HM. In addition, KLK1 and IGK levels at 2 h post-HM were positively correlated.

**Conclusion:**

Our study demonstrated that the salivary proteome is regulated, and antimicrobial proteins are suppressed post-HM. These results suggest that oral immunity was transiently suppressed post-HM. The positive correlation of each protein at 2 and 4 h post-HM suggests that the suppressed state was similarly regulated up to 4 h after a HM. The proteins identified in this study may have applications as stress markers for recreational runners and individuals who perform moderate to HIE on a regular basis.

## Introduction

Moderate exercise, such as 30 min of walking, daily running, and regular sports, has been reported to reduce the risk of upper respiratory tract infections (URTI) (Peters, 1997; Nieman et al., 2011; Bigley et al., 2013; Antunes et al., 2016). Moderate exercise decreases inflammatory cytokines and oxidative stress and improves the function of various immune cells in the resting state (Peters, 1997; Abd El-Kader & Al-Shreef, 2018). On the other hand, prolonged and transient high-intensity exercise (HIE) and training, such as marathons or triathlons, may suppress the immune system and increase the risk of developing URTI (Nieman et al., 1990; Fitzgerald, 1991; Heath et al., 1991). The risk of URTI and lower respiratory tract infections after HIE is higher in healthy individuals than in athletes (Cantó et al., 2018), and it has been reported that there is no difference in risk between males and females (Gleeson et al., 2011; Cantó et al., 2018). The open-window theory explains the temporary immunosuppression caused by high-intensity or extensive exercise that leads to exhaustion. The risk of URTI due to immunosuppression of exercise intensity has also been explained using the J-curve model (Nieman, 2000). A comparison of blood component data before and after marathons showed a decline in the quantitative and qualitative function of immune cells responsible for innate immunity, such as lymphocytes, natural killer (NK) cells, and macrophages, as well as a decline in the function of cellular immunity between 3 and 72 h after participation in a marathon (Nieman & Wentz, 2019). Regarding blood components during immunosuppression by HIE, it has been reported that there is no difference between males and females in plasma immunoglobulin A (IgA), immunoglobulin G and immunoglobulin M concentrations, total white blood cell count, neutrophil count, monocyte count, and lymphocyte count (Gleeson et al., 1999). On the other hand, it has been found that there are differences between men and women in salivary flow rate, IgA concentration, IgA secretion, B cell count, and NK cell count (Gleeson et al., 2011). Depending on the exercise intensity, immunosuppression after HIE has been observed for 2–24 h after exercise, and recovery to the pre-exercise state occurs for approximately two to three days (Fitzgerald, 1991; Kakanis et al., 2010). HIE causes both systemic and local immunosuppression (*e.g.*, in the oral cavity, lungs, bronchial tubes, and skin) after exercise. On the other hand, participation in a half marathon (HM) or middle intensity exercise (not sufficient to induce systemic immunosuppression) has been reported to cause muscle injury and systemic and local immunosuppression (Briviba et al., 2005; Lippi et al., 2011).

The oral cavity is the first frontal region where bacteria and viruses encounter the body and plays an important role in preventing infection. HIE decreases the secretion of saliva and antimicrobial substances (Mackinnon & Jenkins, 1993; Nieman et al., 2002). Saliva is secreted by the parotid, submandibular, and sublingual glands and numerous minor salivary glands (Edgar, 1992). When saliva is supplied to the oral cavity, it provides mucin, lactoferrin, IgA, lysozyme, and lactoperoxidase, which have been reported to contribute to antibacterial activity (Amerongen & Veerman, 2002). Salivary volume and secretion of salivary components are controlled by the autonomic nervous system (Proctor & Carpenter, 2007). Exercise affects saliva secretion by activating the parasympathetic nervous system, thereby regulating the salivary glands. Short-term exercise increases salivary secretory IgA (sIgA), whereas strenuous exercises, such as interval training, marathons, and cycling races, have been reported to decrease salivary flow and sIgA secretion (Mackinnon & Jenkins, 1993; Nieman et al., 2002; Klentrou et al., 2002; Laing et al., 2005). IgA is a glycoprotein produced by mature B cells in blood (Macpherson et al., 2008). It is secreted into bodily fluids such as tears and saliva, as well as bronchial, nasopharyngeal, intestinal, and urogenital secretions (Klentrou et al., 2002). IgA is composed of two heavy chains and two light chains (LC). The immunoglobulin heavy constant alpha 1 (IGHA1) gene encodes a constant region of immunoglobulin heavy chain. The immunoglobulin kappa light chain (IGK) and immunoglobulin lambda light chain (IG*λ*) genes encode a constant region of immunoglobulin LC (Woof & Russell, 2011). IgA can form dimers, which are stabilized between the heavy chains of each monomer and joining site and the J chain (Woof & Russell, 2011). Salivary IgA is used as a mental and physical stress marker (Pedersen & Hoffman-Goetz, 2000). The reported roles of IgA include the inhibition of bacterial aggregation, neutralization of viruses, and opsonization of support (Kaetzel et al., 1991; Walker, 2004; Fábián et al., 2012).

Decreased sIgA secretion due to high training load is thought to be associated with an increased risk of respiratory disease (Fahlman & Engels, 2005; Neville, Gleeson & Folland, 2008; Walsh et al., 2011; Leicht et al., 2012). Previous studies on the immunosuppressive state induced by exercise have mainly conducted in male for used the proteome and metabolome of blood components. Consequently, the detailed behavior of blood metabolites has become clearer (Kurgan et al., 2019; Guseh et al., 2020). However, studies on the oral cavity have been confined to quantifying salivary volume, protein concentration, and marker proteins to easily determine the body’s immune status. Few studies have quantitatively analyzed salivary proteins before and after the immune suppression state in the oral cavity, which is the frontline of defense response to pathogen infection, and studies on female have been limited. The primary rationale for the predominant inclusion of male participants in exercise-related studies is the perceived impact of female sex hormones on a multitude of physiological and psychological systems (Oosthuyse & Bosch, 2010). However, research has produced inconsistent findings regarding the involvement of female hormones in saliva secretion and composition, with some studies indicating a relationship (Ship, Patton & Tylenda, 1991) and others demonstrating no such effect (Wilson, Lorenz & Heiman, 2018). Therefore, further investigation is necessary to elucidate the precise nature of this relationship.

In this study, conducted on non-athletic females without consideration of menstrual cycles, we performed a quantitative proteomic analysis of saliva in the immunosuppressed state (*i.e.,* decreased salivary outflow, increased salivary protein concentration, and decreased IgA per unit protein) in the oral cavity induced by a HM and tested the correlation of regulated proteins. The results provide insight into the local stress state induced by a HM for female. Furthermore, this insight into local stress responses may contribute to the health management of female athletes and amateur recreational runners temporarily exposed to exercise stress.

## Materials & Methods

### Participants and experimental design

**Figure 1:**
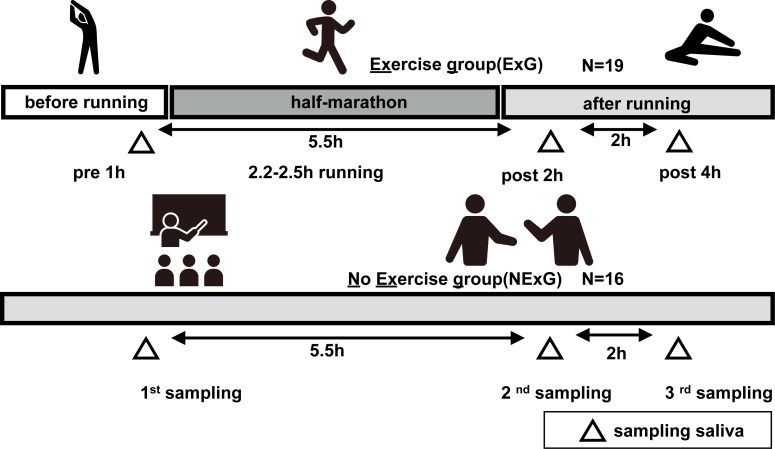
Study diagrams and sampling points of saliva. The exercise group (ExG) that performed the half marathon (HM) and non-exercise group (NExG) consisted of 19 and 16 participants, respectively. Salivary samples in the ExG were collected 1 h pre-HM and 2 h post and 4 h post-HM, while those in the NExG were also collected at three intervals, 5.5 h and 7.5 h after the first sampling. The intervals between the effects of HM on the secretion rate, protein concentration, and IgA secretion in saliva were approximately the same. (The pictogram copyright; https://www.silhouette-illust.com/illust/43300, 46868, 28576, 28492, 42427).

In the exercise group (ExG), nineteen healthy female university students participated in HM race held in Shibetsu City, Hokkaido (44°12′11″N, 142°27′37″E) in 2018. The participants were volunteers with no known underlying diseases or predisposing factor to infection. They trained for approximately two months prior to the HM, including running approximately 5–10 km a week, for endure the HM race. The median and whole range of age, weight, body mass index (BMI), HM completion time, race pace and energy consumption during pre HM to post 2 h HM of the participants were shown in Table 1. In the non-exercise group (NExG), sixteen healthy female university students had not participated in the ExG. The sampling of the NExG was performed during the time they attended lectures at the university (Fig. 1). It should be noted that menstrual cycles were not considered for either the ExG or NExG. The median and whole range of age, weight, BMI and energy consumption during 1st to 2nd sampling were shown in Table 1.

The ethics committee of the Nayoro City University approved the experimental protocol (approval number 17-22). All participants gave signed informed consent.

### Saliva sampling

Salivette Cotton (Sarstedt K. K, 51.1534; Sarstedt Group, Starstedt, Germany) was utilized for saliva collection. Participants were instructed to rinse their mouths with water for a duration of 5 min prior to sampling. At the sampling time, the patients placed a cotton roll for in their mouths for a period of 2 min. The ExG underwent sampling at 1 h prior to, 2 h post, and 4 h post-HM. In contrast, the NExG underwent sampling was performed at 10:00, the second at 14:30, and the third at 18:30 as depicted in Fig. 1. The intervals between salivary collections for both the ExG and NExG were comparable. Ad libitum water consumption was permitted for participants in both groups. Saliva samples were fixed in liquid nitrogen and stored at −80 °C until analysis.

**Table 1:** Subject characteristics. HM energy consumption represented energy consumption during HM in ExG. Energy consumption represented total amount of pre-HM to 2 h post-HM in ExG and 1st to 2nd sampling in NExG. Data show median, maximum and minimum value. The *p*-values refer to the comparison between the NExG and ExG using Mann-Whitney rank test. The power (1-β err prob) show *post hoc* analysis by G-power software. BMI, Body Mass Index.

	Median (min–max)			
	Ex group (ExG) *N* = 19	NEx group (NExG) *N* = 16	*p* value	Power (1-β err prob)
Age (years)	20.4 (19.1–21.4)	20.1 (18.1–22.5)	0.960	0.055
Body mass (kg)	53.1(44.8–67.0)	49.5 (44.2–68.2)	0.689	0.050
Length (cm)	159 (151–177)	156 (147–178)	0.430	0.108
BMI (kg/m^2^)	20.96 (19.65–22.34)	20.52 (19.65–22.34)	0.401	0.054
Running time (min)	135.05 (107.20–148.78)	–	–	–
Race pace (m/min)	159.8 (142.50–197.10 )	–	–	–
HM Energy consumption (Kcal)	1152.5 (969.4–1583.0)	–	–	–
Average HM Energy consumption (Kcal/min)	8.58 (6.97–12.72)	–	–	–
Total Energy consumption (Kcal)	1705.12 (1445.30–2419.98)	588.55 (537.87–793.83)	<0.001	1.000

### Analysis of saliva volume and concentration

Saliva volume was determined by weighing the pre collection tube and post collection tube assuming a density of 1 g/mL. Salivary flow rates were calculated by dividing the volume of saliva by the collection time. Protein concentration was quantified using the Bradford assay method (Bradford, 1976). The assay was performed using Bradford Protein Assay Kit (TAKARA, T9310A, Shiga, Japan).

### SDS-PAGE, Silver Staining and CBB staining

The saliva protein was suspended in 2x SDS sample buffer (200 mM Tris–HCl pH 6.8, 400 mM DTT, 8% SDS, 0.4% bromophenol blue, 40% glycerol), and swept on the 10–12.5% acrylamide gel and separated by sodium dodecyl sulfate-polyacrylamide gel electrophoresis (SDS-PAGE) under reducing conditions. The silver staining was then performed using a silver staining kit (291-5030, WAKO, Osaka, Japan) according to the manufacturer’s protocol. The coomassie brilliant blue (CBB) staining were used as a loading control. CBB staining performed using a EzStain Aqua (AE-1340; ATTO, Tokyo, Japan) according to the manufacturer’s protocol.

### Protein preparation and iTRAQ labeling

Salivary proteins from nine ExG participants were used for the iTRAQ analysis. The iTRAQ technology for protein quantitation using mass spectrometry is a recent, means of determining relative protein levels in up to 4–8 samples simultaneously (Ross et al., 2004). A total of 5 µg of each protein was subjected to trypsin treatment using a captured trypsin column (635722; TaKaRa, Shiga, Japan). After trypsin treatment, the samples were run on a mono-spin C18 column (5010-21732, GLS, Tokyo, Japan). The digested peptides were labeled with iTRAQ reagent according to the manufacturer’s protocol (Applied Biosystems). In brief, the digested peptides were mixed with iTRAQ reagents (AB Sciex, 4352135, CA, USA) as follows: 1 h pre-HM (tag 114 or 116) and 2 h post HM (tag 115 or 117). The mixed samples were incubated at 24 °C for 1 h, and then the pooled samples 114, 115, 116, and 117 were further pooled into one tube. Each sample was treated with an SCX column (5010-21726; GLS, Tokyo, Japan) and eluted with a cation exchange buffer (10 mM KH_2_PO_4_, 25% acetonitrile). The eluted solution containing the peptides was dried using a vacuum concentrator. The precipitate was dissolved in a solution (2% acetonitrile and 0.1% formic acid) and loaded onto an Eksigent microLC 200 (Eksigent, Dublin, CA, USA) equipped with an electrospray ionization triple quadrupole-linear ion trap system (Triple Quad 5500+LC-MS/MS system; SCIEX, Framingham, MA, USA). iTRAQ data were analyzed using ProteinPlot™ software ver 5.0.2 (SCIEX). The SwissProt database containing 20,411 human sequences, was used for protein verification.

### Western blotting

Salivary samples were centrifuged for 5 min at 12,000 g at 4 °C before SDS-PAGE. Western blotting (WB) was performed as previously described (Lüllau et al., 1996). Briefly, 3 µg of salivary protein was loaded onto 10–12.5% SDS acrylamide gels and separated for 80 min at a constant current 30 mA. Following SDS-PAGE, proteins were transferred to PVDF membranes (IEVH00005; Merck Millipore, Tokyo, Japan) and blocked for 1 h with a 2.5% non-fat milk solution in PBS containing 0.1% Tween. Subsequently, primary antibodies against IGHA1 (Monoclonal rabbit 1:5000 dilution; 31-1030-00; RevMab Biosciences, South San Francisco, CA, USA), Cystatin S (CST4) (polyclonal rabbit 1:5000 dilution; GTX100690; GeneTex, Irvine, CA, USA), IGK (Polyclonal rabbit 1:2000 dilution; 14678-1-AP; Proteintech Group, Inc, Chicago, IL, USA), Zinc Alpha 2 Glycoprotein (ZA2G) (polyclonal rabbit 1:5000 dilution; AP6628a; Abcepta, CA, USA), and Kallikrein 1 (KLK1) (polyclonal rabbit 1:3000 dilution; PA005798; Cusabio Technology LLC, Houston, TX, USA) were applied and probed. The specific proteins were then detected using with horseradish peroxidase (HRP) conjugated secondary antibodies (1:5000 dilution; NA934, GE Healthcare, 1:5000 dilution; NA931, GE Healthcare, Tokyo, Japan), developed with Immobilon Western Chemiluminescent HRP Substrate (WBKLS0100; Merck Millipore, Tokyo, Japan), and visualized utilizing the LumiVision PRO 400EX system (AISIN, Aichi, Japan).

### Statistical analysis

Data are presented as medians with interquartile ranges or whole ranges, as appropriate. All data were analyzed as normally distributed. Age, height, weight, BMI, and total energy consumption were evaluated using Mann–Whitney U-tests. These values were subjected to a post hoc test using G-Power software (Faul et al., 2007). The Shapiro–Wilk test was used to analyze the normality of salivary volume, protein concentration, iTRAQ, and WB data. For comparison of baseline values and other values (*e.g.*, pre HM and 2 h post-HM; 1st sample, and 2nd sample) of ExG and NExG, we used the Wilcoxon signed-rank sum test. Statistical significance was set at *p* < 0.05. Statistical analyses were performed using the SPSS software (version 23.0; IBM Corp., Armonk, NY, USA). Spearman’s rank correlation coefficient test was performed to determine the correlation of regulated proteins pre and post-HM. Boxplots were generated using BoxPlotR (Spitzer et al., 2014). A Heatmap was plotted using http://www.bioinformatics.com.cn/srplot, an online platform for data analysis and visualization.

## Results

### HM led to decreased salivary secretion and IGHA1

**Figure 2:**
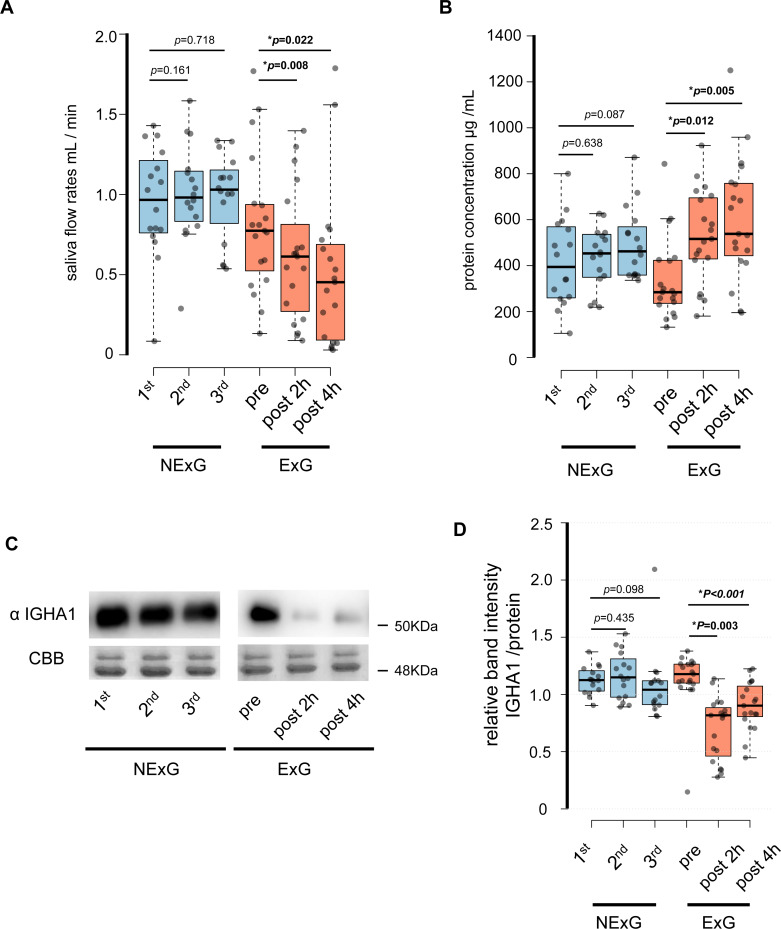
Effect of half-marathon on saliva secretion rate, protein concentration, and IGHA1 secretion. (A) The secretion rate of saliva in the non-exercise group (NExG, *N* = 16) and exercise group (ExG, *N* = 19). (B) The salivary protein concentration in the NExG and ExG. (C) Western blot analysis of salivary IGHA1. CBB staining was used as a loading control. (D) The relative band intensity of salivary IGHA1 in the NExG and ExG. The band intensity from first sampling and pre HM was compared with those from second and third samplings in the NExG, and 2 h post and post 4 h HM, respectively. The fold changes were considered as 1.0 at first sampling in the NExG and pre HM in the ExG, respectively. An asterisk (*) indicates significant difference at *p* < 0.05 level.

In this study, 19 participants took part in an HM as the ExG and 16 participants did not take part in the HM and were the NExG (Fig. 1). A post hoc analysis was conducted to estimate statistical power, utilizing the obtained sample size and a predetermined *α* level of 0.05 for variables including age, body mass, length, BMI, and total energy consumption (Table 1). The statistical power of age, body mass, length, and BMI were found to be less than 0.2, while the statistical power of total energy consumption was determined to be greater than 0.8 (Table 1). In the ExG, salivary secretion was reduced, and the amount of salivary proteins increased post-HM compared to those observed pre-HM (Figs. 2A and 2B). The median and *p* values for salivary secretion were 0.61 mL/min (*p* = 0.008) and 0.45 mL/min, (*p* = 0.022) at 2 and 4 h post-HM, respectively. The amount of salivary proteins was 516 µg/mL, (*p* = 0.005) and 538 µg/mL, (*p* = 0.012) at 2 h and 4 h post-HM, respectively. There were no differences in salivary volume and protein concentration in the NExG for the 2nd and 3rd sampling intervals compared with the 1st sampling saliva (Figs. 2A and 2B). The relative amounts of salivary IGHA1 from 2 and 4 h post-HM were decreased compared with pre-HM. The median and *p* values were 0.82 and *p* = 0.003 at 2 h post-HM, respectively, and 0.90 and *p* < 0.001 at 4 h post-HM, respectively. There were no differences in the relative amounts of IGHA1 in NExG at the three sampling intervals (Fig. 2D).

### Proteomic analysis of salivary proteins before and after exercise

**Figure 3:**
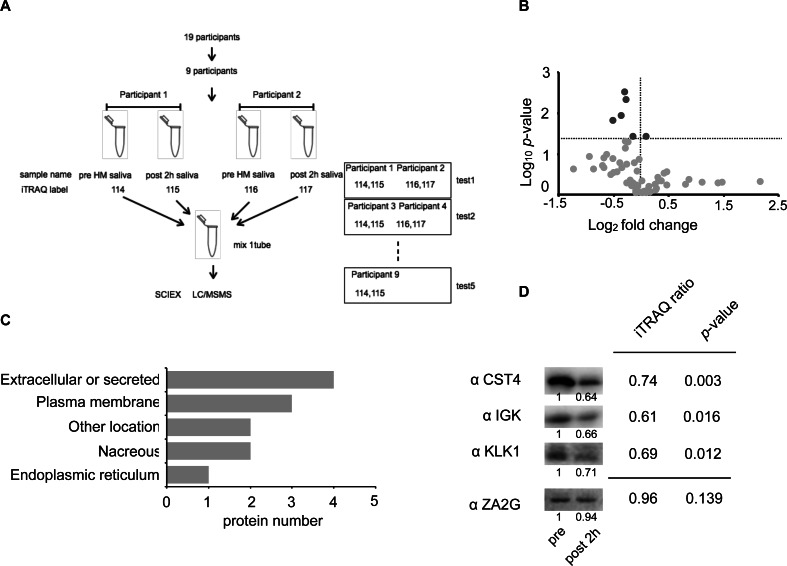
Identification of the half-marathon regulated salivary proteins by iTRAQ analysis. (A) Schematic diagram of the iTRAQ based on experiments. The salivary proteins of nine participants from pre- and post 2 h HM were analyzed on five separate occasions. Four samples were pooled into one sample for either pre-HM and 2 h post-HM. (B) Protein distribution in accordance with log_10_ *P*- *versus* and log_2_ ratio of the detected 1 ≤ peptide proteins. The darkgray dot indicated the salivary proteins that changed 2 h post-HM compared with pre-HM (*p* < 0.05). (C) Subcellular annotation of differentially expressed proteins in saliva. The subcellular location was shown by UniProt annotation. (D) Western blot profile for CST4, IGK, KLK1, and ZA2G using iTRAQ samples. The numbers below the blot indicate the relative signal intensity of each protein compared with pre-HM sample.

To consider the changes in salivary proteins pre and post-HM in the ExG, we compared salivary proteins separated by SDS-PAGE and performed silver staining (Fig. S1). In ExG, the protein bands in the vicinity of 15, 30, and 50 kDa decreased in the salivary samples collected 2 and 4 h post-HM compared with pre-HM (Fig. S1). In contrast, no differences in the band intensities of salivary proteins were found at the three sampling intervals of NExG (Fig. S1). To analyze the protein changes, focusing on the early stages of immunosuppression, we performed quantitative proteomic analysis using iTRAQ labeling and examined salivary proteins 1 h pre and 2 h post-HM in nine participants (Fig. 3A).

We identified 4,089 proteins, as shown in Fig. S2, and our iTRAQ data were compared with the protein expression data available through the Human Salivary Protein Wiki (HSP-Wiki: https://www.salivaryproteome.org/public/index.php/Main_Page) (Lau et al., 2021). 1,039 proteins were matched with the Human Salivary Proteome Wiki data. 60 proteins were identified as 1 ≤ peptides (Table S1 and Fig. 3B), and six proteins were significantly different between 1 h pre and 2 h post-HM (*p* < 0.05) (Fig. 3B). We were also able to identify four proteins that were suppressed less than 0.8-fold (Table 2), while no proteins that were expressed more than 1.2-fold could be identified.

**Table 2:** The salivary proteins regulated by half marathon. The fold change shows the relative ratio of 2 h post-HM to pre-HM. The *p* values shows raw *p* values. The peptide shows the unique peptide number detected in analyses samples. An asterisk (*) indicates significant difference at *p* < 0.05 level.

Accession	Description	Gene name	MW (KDa)	Fold change (FC)	log2 FC	Peptide	*p* value
P01036	Cystatin S	*CST4*	16.21	0.74	−0.43	4	0.003
P01876	Immunoglobulin heavy constant alpha 1	*IGHA1*	37.65	0.76	−0.40	7	0.005
P06870	Kallikrein 1	*KLK1*	28.89	0.69	−0.54	4	0.012
P0DOX7	Immunoglobulin kappa light chain	*IGK*	23.37	0.60	−0.74	6	0.016

The factors that were suppressed by less than 0.8-fold between 1 h pre and 2 h post-HM were CST4, IGHA1, KLK1, and IGK. The fold change and *p* values for CST4 were 0.74 and *p* = 0.003, respectively. The fold change and *p* values for IGHA1 were 0.76 and *p* = 0.005, respectively. The fold change and *p* values for KLK1 were 0.69 and *p* = 0.012, respectively. The fold change and *p* values for IGK were 0.60 and *p* = 0.016, respectively (Table 2 and Fig. 3B). IGHA1 has been used as a suppression marker of salivary immunity, and similar results were obtained by WB and iTRAQ analyses (Figs. 2C and 2D).

Based on the results of the iTRAQ experiments, we immunoblotted four proteins: CST4, IGK, KLK1, and ZA2G. ZA2G was a control marker with constant expression, whereas the other three proteins were suppressed as variable factors (Fig. 3D).

### Subcellular and functional characterization of the candidate proteins

Among the 48 factors (peptides ≥ 1), 16 were classified as secretory proteins (Table S1). Ten factors (*p* < 0.05) were classified based on UniProt annotation (https://www.uniprot.org/). Four proteins were classified as extracellular or secreted, three as plasma membrane, two as nacreous, and two belonged to other locations (Fig. 3C).

### IGK, CST4, and KLK1 were suppressed by the HM

**Figure 4:**
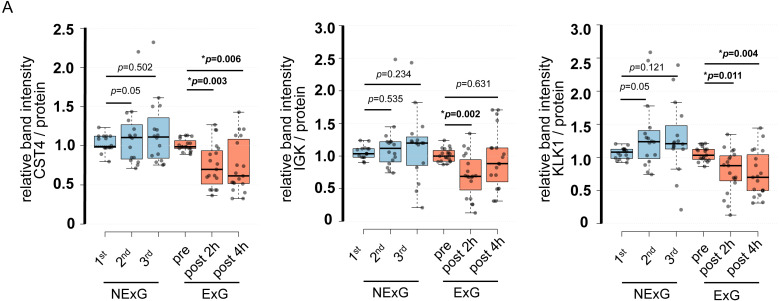
Confirmation of iTRAQ ratio by western blot analyses. (A) The expression of CST4, IGK and KLK1 in saliva. The center lines show the medians of the data in the ExG (*N* = 19) and NExG (*N* = 16); box limits indicate the 25th and 75th percentiles as determined by R software; whiskers extended 1.5 times the interquartile range from the 25th and 75th percentiles; outliers are represented by dots. Differences between pre-and post-HM or first and other time points of the NExG were tested using the Wilcoxon signed-rank test. An asterisk (*) indicates significant difference at *p* < 0.05 level.

To further validate the iTRAQ results, we examined protein expression levels by WB. WB was performed using saliva collected from the ExG and NExG. All three proteins (CST4, IGK, and KLK1) were suppressed in the ExG 2 h post-HM. CST4 and KLK1, but not IGK, were suppressed 4 h post-HM (Fig. 4). Median and *p* values for each of the proteins were as follows: 2 h post-HM: IGK (0.69, *p* = 0.002), KLK1 (0.85, *p* = 0.011), and CST4 (0.70, *p* = 0.003); 4 h post-HM: CST4 (0.62, *p* = 0.006) and KLK1 (0.70, *p* = 0.004). These results indicated that the iTRAQ ratios were consistent with the quantitative results obtained by WB analysis. In the NExG, CST4 and KLK1 were upregulated at 2nd time samples compared with 1st time samples. The median and *p* values were 1.09 and *p* = 0.05 for CST4 and 1.23 and *p* = 0.05 for KLK1, respectively (Fig. 4). No significant differences were found outside of these factors at each sampling point in the NExG.

### Correlation of IGHA1, IGK, CST4, and KLK1

**Figure 5:**
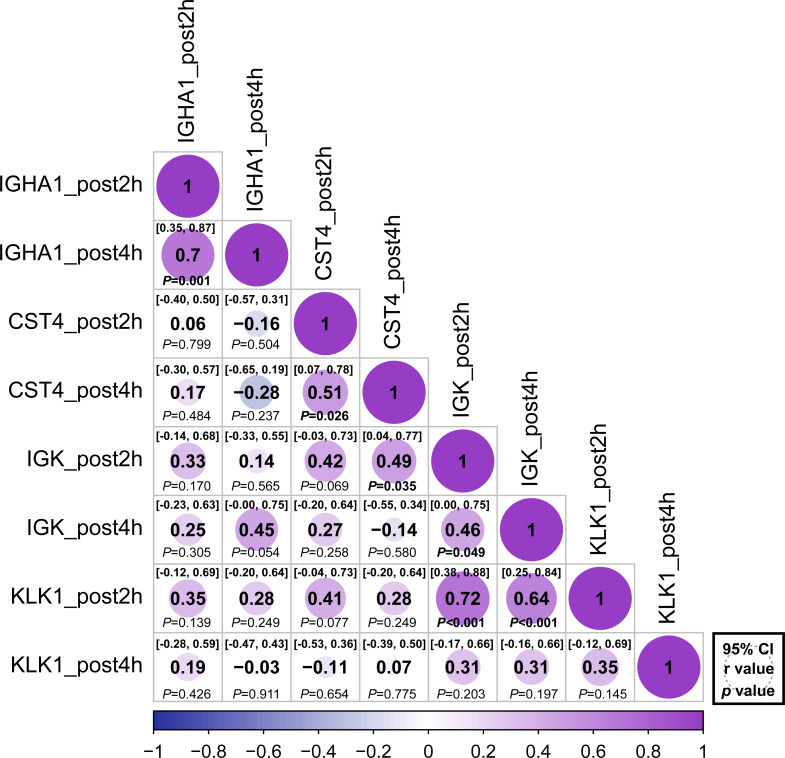
Correlation matrix of different expression protein. Correlations between the relative abundance of IGHA1, CST4, IGK and KLK1 at 2 h post and 4 h post-HM. Spearman correlation coefficients (*r*) and 95% confidence interval (95% CI) were shown for each protein. The bold font *p* - value was shown for significant difference at *p* < 0.05 level.

The relative abundance of protein changes in the ExG was tested using Spearman’s correlation conflict test. The relative abundances of IGHA1, CST4, and IGK were positively correlated 2 h and 4 h post-HM (Fig. 5). Similarly, a positive correlation was observed between KLK1 protein abundance at 2 h post-HM and IGK protein abundance 2 h and 4 h post-HM, and between CST4 protein abundance at 4 h post-HM and IGK protein abundance 2 h post-HM (Fig. 5). In contrast, no relationship was observed between other protein abundances and the time points (Fig. 5).

## Discussion

The findings of this study reveal the salivary proteome during HM stress. After 2 h, HM stress suppressed salivary antimicrobial proteins such as KLK1, CST4, and IGK in association with IGHA1. After 4 h, HM, IGHA1, KLK1, and CST4, but not IGK, were suppressed after 2 h post-HM.

In addition, the relative abundances of IGHA1, CST4, and IGK were positively correlated 2 h and 4 h post-HM. Similarly, KLK1 levels at 2 h post-HM were positively correlated with IGK at 2 h and 4 h post-HM timepoints. The suppression of these antimicrobial proteins suggests that HM suppresses local immunity in the oral cavity and the suppression lasts from 2 h to 4 h post-HM. Our findings will help improve our understanding of local stress responses in the oral cavity due to exercise stress.

In the ExG, HM running time and race pace were comparable to those reported for women participants (*N* = 2,852) in the 2017 HM race in Ljubljana, Slovenia (Nikolaidis, Ćuk & Knechtle, 2019). It was thought that when ExG ran a HM, the energy load was similar to that of a typical recreational runner.

We also analyzed the IGHA1 secretion rate, saliva secretion per unit of time, and saliva concentration as immunosuppression markers. The results suggest the induction of suppression of local immunity in female university students without general exercising habits. In contrast, there were no changes in the saliva flow rate and concentration or IGHA1 levels of university students taking classes in the normal state (Fig. 2). For quantitative proteomic analysis, we performed iTRAQ analysis. iTRAQ is widely used in proteomics research as a quantitative proteomic technique; it has relatively high sensitivity and can identify a large number of proteins compared to traditional proteomic approaches (Ross et al., 2004). The iTRAQ results, using saliva 2 h post-HM, showed that six factors with 1 ≤ peptides and *p* < 0.05 were suppressed post-HM compared to their pre-HM levels, and the remaining 54 factors were unchanged (Table 2, Table S1).

The candidate proteins IGHA1, IGK, CST4, and KLK1 play a role in antibiotic activity and stress response stimulation. The antimicrobial factors IGHA1 and IGK are components of IgA and are involved in immune responses in serum and saliva. IGK, which is comprised of immunoglobulin, is the light chain (LC) which is involved in antigen recognition and activation of neutrophils and neurons (Brebner & Stockley, 2013). CST4 is a cysteine protease that plays a protective and regulatory role in the oral cavity (Dickinson, Thiesse & Hicks, 2002). CST4 has been reported to exert an antibacterial effect on *Porphyromonas gingivalis,* which is involved in periodontal disease (Blankenvoorde et al., 1996). Similarly, salivary KLK1 reportedly degrades neisserial heparin-binding antigen, a lipoprotein that is exposed on the surface of the meningococcal cell membrane (Pantano et al., 2019).

KLK1 may be involved in stress response stimulation. KLK1 is a serine protease that is a component of the kallikrein kinin system (KKS) (Yiu et al., 2014). KLK1 cleaves low-molecular-weight kininogens into kinins, which perform biological functions *via* kinin receptor signaling (Kakoki & Smithies, 2009). KKS is involved in inflammation, coagulation, pain, and vascular permeability *via* kinin production. It is assumed that the suppression of these proteins after HM means that antimicrobial activity is suppressed, and the capacity to adapt to the stress response is reduced.

In contrast, the salivary protein response may adapt to exercise stress by being suppressed. Asthmatics experience bronchoconstriction-induced bradykinin inhalation, but healthy subjects do not (Polosa et al., 1994). The effects of bradykinin in healthy subjects under exercise stress are unknown, but it is hypothesized that if KLK1 affects bronchioles as well as the oral cavity, suppression of KLK1 may be related to bronchoconstriction during excessive exercise and may be involved in respiration.

However, to date, KLK1 behavior and mechanism during exercise stress have not yet been reported. Our study showed that KLK1 was suppressed under HM stress. The relative changes and protein functions of IGHA1, IGK, CST4 and KLK1 suggest that HM suppresses antimicrobial activity in the oral cavity.

Saliva is an indicator of mental and physical stress. IGHA1 has been used as a marker of physical and mental stress (Pedersen & Hoffman-Goetz, 2000). Moderate exercise increases sIgA, as does the activity of immune factors in the blood, while it decreases sIgA when the immune system is suppressed by HIE, such as during marathons (Nieman & Wentz, 2019). In terms of mental status, continued anxious mental state or transient nervousness decreases sIgA levels. On the other hand, happy feelings and positive thoughts have been reported to increase sIgA levels (Obayashi, 2013). Our results show that IGHA1, IGK, CST4, and KLK1 levels were similarly suppressed after HM. A recent study reported that CST4 was activated and IGHA1 was suppressed during acute social stress in healthy young adults (Zallocco et al., 2021). In addition, salivary proteins from highly trained athletes before and after competition increase the secretion of CST4 as a subacute response to aerobic and anaerobic exercises (Sant’Anna et al., 2019). In this study, CST4 and KLK1 levels were also increased in NExG that attended university lectures. These findings suggest that CST4 is increased by moderate exercise and mental stress and decreased by physical stress, such as exhaustion.

Heaney et al. (2018) reported that salivary IGK and IG*λ* levels were suppressed after short-term intense training among highly trained male cyclists. In addition, the effects of exercise on salivary free LC secretion rates appear to be dependent on age; in elderly participants, significant reductions in salivary free LC have been observed post-exercise, whereas only modest reductions occurred in the young (Heaney et al., 2016). Considering that the amount of LC secretion under physical stress varies with age, IGK returning to the pre HM state level 4 h post-HM compared to other factors may be age-related. As a cause of the lack of suppression of IG*λ* levels in this study compared to studies of male cyclists, there are some likely causes for the differences between the study groups, age, sex, and exercise levels. Suppression of IGHA and IGK, components of IgA, may be involved in IgA function, and changes in specific LCs may imply the regulation of inflammatory responses. Although the changes in IgA secretion and its involvement in the immune suppression state have been discussed (Campbell & Turner, 2018), to clarify the local immunity in the oral cavity and saliva in more detail, it would be helpful to examine not only the total amount of IgA but also its composition and activity.

To the best of our knowledge, there are no studies reporting change in IGK due to exercise and mental condition. Similarly, there have been no reports of exercise or mental stress being associated with KLK1. The results of this study and previous studies suggest that CST4 is activated during mental stress and suppressed during physical fatigue. While sIgA is suppressed during both mental stress and physical stress, CST4 may respond differently to mental stress and physical stress. Analysis of IGHA1 and CST4 behavior in unknown saliva samples may provide a means of understanding mental and physical stress conditions.

In this study, IGHA1, CST4, IGK, and KLK1 were detected in saliva and were found to be regulated by HM stress. Data from the Human Protein Atlas (http://www.proteinatlas.org) highlighted that the protein expression of IGHA1 has been reported in the intestine, lymphoid tissue, salivary glands, and stomach. Similarly, CST4 expression has been reported in the salivary gland, IGK expression in the intestine and lymphoid tissue, and KLK1 expression in the pancreas and salivary glands.

We performed correlation analysis to determine whether there was a relationship between the regulated proteins (Fig. 5). Positive correlations were observed 2 and 4 h after HM for IGHA1, IGK, and CST4. These results suggest that protein regulation continued at 2 and 4 h post-HM. On the contrary, there was no correlation between IGHA1 and other proteins, suggesting that IGHA1 and other proteins might be regulated through different pathways by HM stress. IgA has been utilized as an indicator of immunosuppression prior to and following physical exertion, yet there may not be as many variables that are similarly governed by IgA-regulated pathways in varied exercise conditions and stress conditions. KLK1 expression after 2 h and IGK expression after 2 h and 4 h were positively correlated (Fig. 5). However a positive interaction between KLK1 and IGK may be difficult to determine because our study did not observe protein localization or expression during HM stress.

Additionally, these findings cannot be extrapolated to all groups. The limitations of this study are as follows: (1) the study was conducted on women of a specific age group, the sample size was small and not considered for menstrual cycles; (2) the study did not include men of the same age group; and (3) the study did not assess exercise stress in detail. Although this study was conducted on exercise stress of completing an HM, the information obtained in the experiment was not sufficient to analyze the exercise stress state in detail, and the detailed exercise stress state of each individual was unclear. Further studies are required to address this issue.

## Conclusions

HM reduces the saliva flow rate and IGHA1 secretion. Proteome and WB analysis showed that CST4 and KLK1 were suppressed 2 h and 4 h post-HM, and IGK was suppressed at 2 h post-HM. In addition, IGHA1, CST4, and IGK proteins were positively correlated at 2 h and 4 h post-HM, suggesting that protein regulation was maintained at 2 h and 4 h post-HM. The findings of this study can be applied to the health management of recreational runners and people who engage in moderate to high intensity physical activity in daily life.

##  Supplemental Information

10.7717/peerj.15075/supp-1 Supplemental Information 1 List of salivary proteins identified by iTRAQ analysis in pre- and post-half marathon The peptide shows unique peptide number in analyses samples. *The subcellular location and tissue specificity shows the localization of detected proteins. Data were from the Human Protein Atlas. n.d; no data. **The mRNA was described as to specifically expressed in any of the salivary glands. The sites of adult salivary glands that were more than twice as active as fetal salivary glands with *P* < 0.05 were shown. ms; submandibular gland, PAR; parotid gland, SL; sublingual gland. The data of mRNA localization were from (Saitou et al., 2020). ***The whole saliva indicates whether the detected protein is contained in the salivary proteins of the Human Salivary Protein Wiki (https://salivaryproteome.nidcr.nih.gov/). Y; contain in saliva, -; no existence.

10.7717/peerj.15075/supp-2 Supplemental Information 2 SDS-PAGE analysis of salivary proteins A total of 3 µg protein was loaded from each sample. The protein samples in the NExG were from the first, second, and third sampling, while those in the ExG were from pre-HM and 2 h post and 4 h post-HM. Protein samples from a single subject were used in NExG and ExG. The protein bands with asterisks in the ExG decreased 2 h post and 4 h post-HM compared with pre-HM.

10.7717/peerj.15075/supp-3 Supplemental Information 3 Venn diagram of the number of quantified proteins and Protein distribution of molecular weight and fold change (A) Venn diagram of the number of quantified proteins derived from the five sets of iTRAQ experiments. (B) Protein distribution in accordance with log_10_ theoretical molecular weight *versus* log_2_ fold change of all the detected proteins of 1 ≤ peptide.

10.7717/peerj.15075/supp-4 Supplemental Information 4 Raw data for the tables and figures

10.7717/peerj.15075/supp-5 Supplemental Information 5 WB raw image

## Additional Information and Declarations

### Data Availability

The following information was supplied regarding data availability: The raw measurements are available in the Supplemental Files.
