# Supplementary figures and images for: Analysis of IGHA1 and other salivary proteins post half marathon in female participants
Source: PeerJ. 2023 May 11;11:e15075. doi: 10.7717/peerj.15075 (PMC10183162; doi:10.7717/peerj.15075)

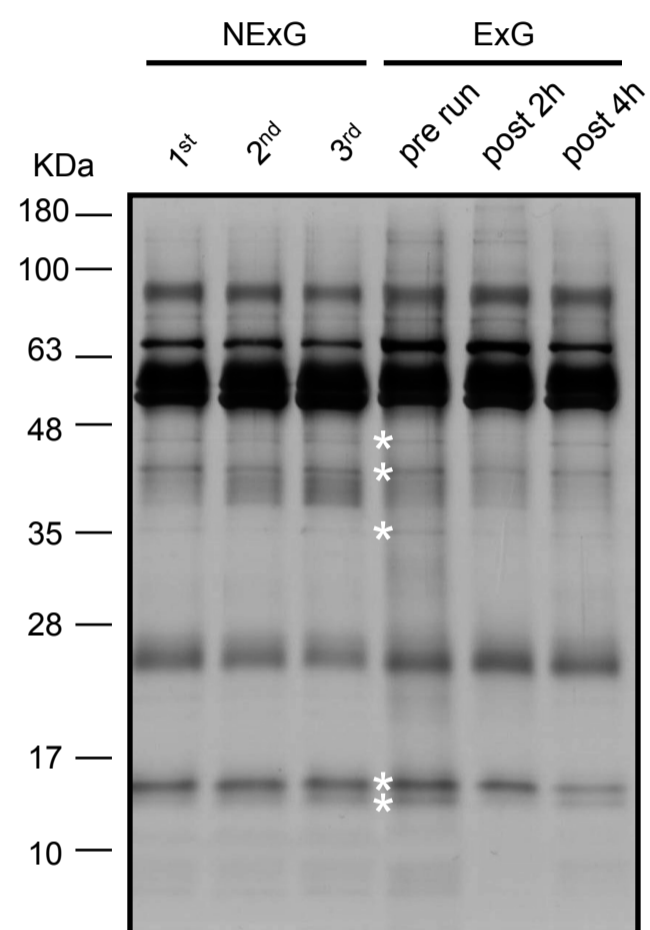

Maruyama et al sFig.1

Supplement: Supplemental Information 2 — A total of 3 µg protein was loaded from each sample. The protein samples in the NExG were from the first, second, and third sampling, while those in the ExG were from pre-HM and 2 h post and 4 h post-HM. Protein samples from a single subject were used in NExG and ExG. The protein bands with asterisks in the ExG decreased 2 h post and 4 h post-HM compared with pre-HM. [file peerj-11-15075-s002.pdf]

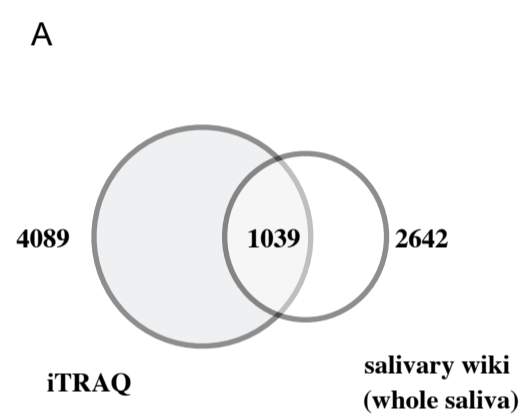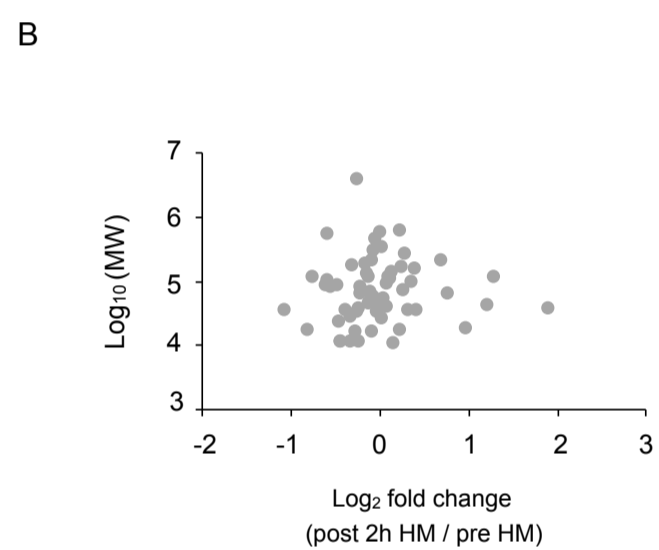

Supplement: Supplemental Information 3 — (A) Venn diagram of the number of quantified proteins derived from the five sets of iTRAQ experiments. (B) Protein distribution in accordance with log10 theoretical molecular weight versus log2 fold change of all the detected proteins of 1 ≤ peptide. [file peerj-11-15075-s003.pdf]

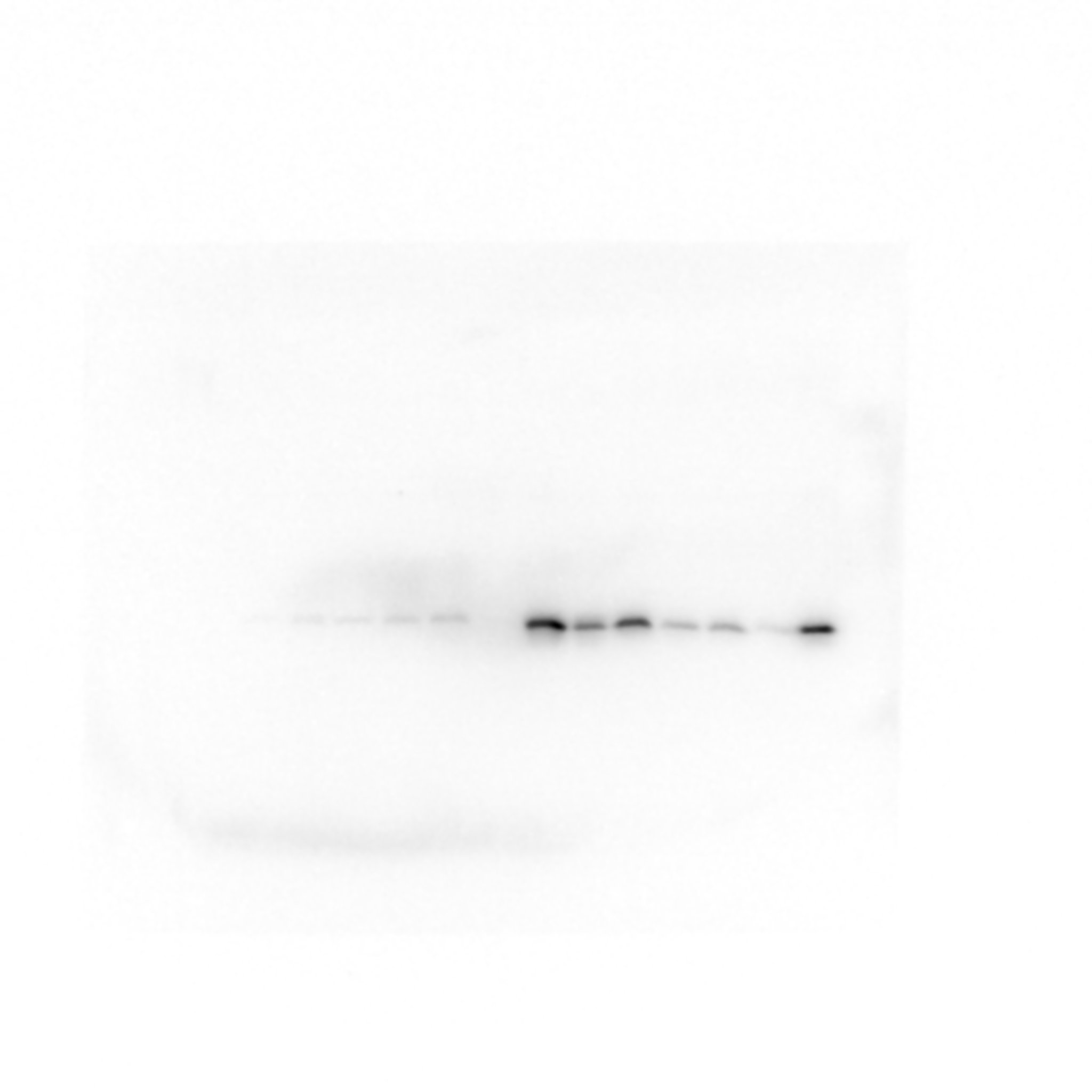

Supplement: Supplemental Information 5 [file peerj-11-15075-s005.zip › WB image/cst4_itraq.jpg]

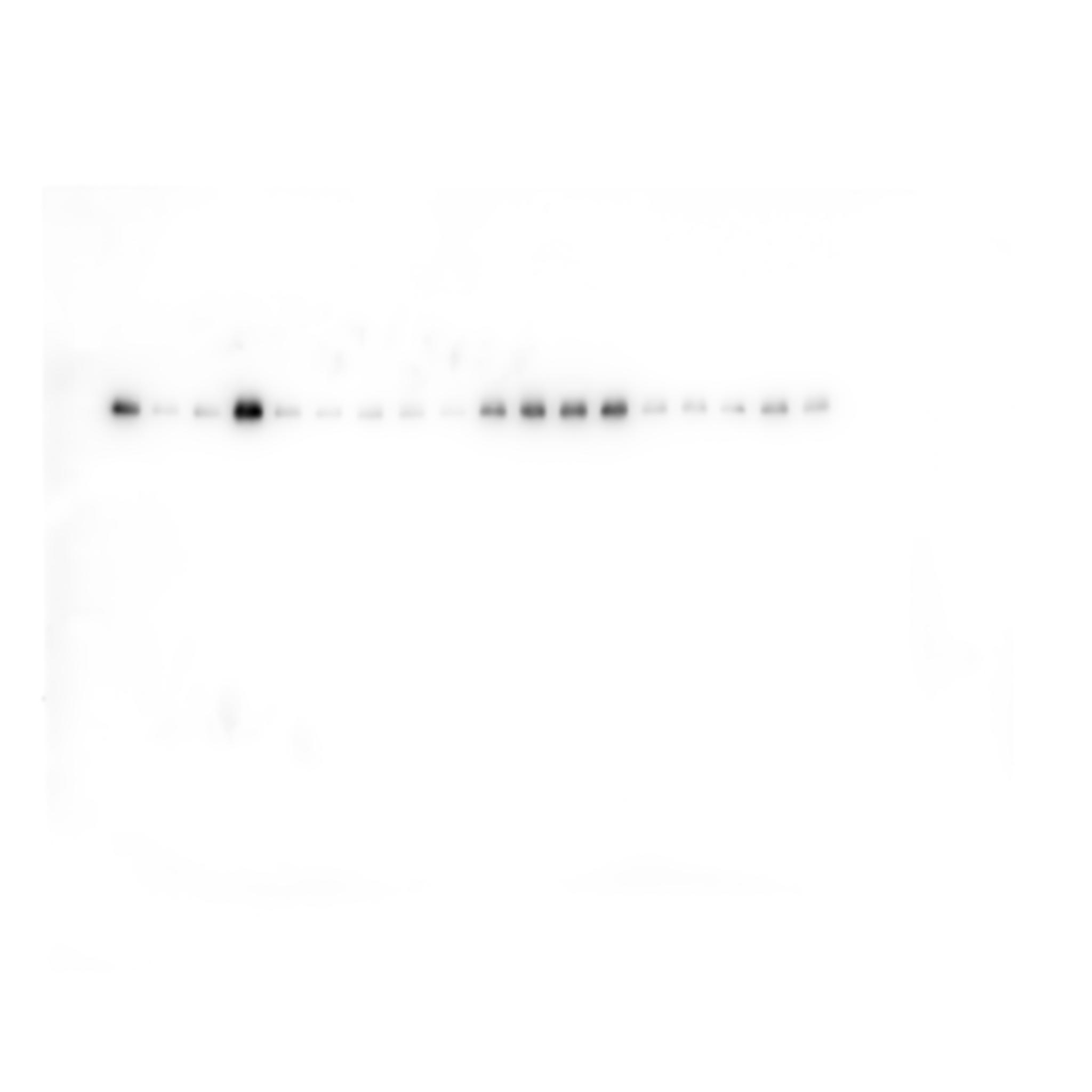

Supplement: Supplemental Information 5 [file peerj-11-15075-s005.zip › WB image/IGHA1_ExG.jpg]

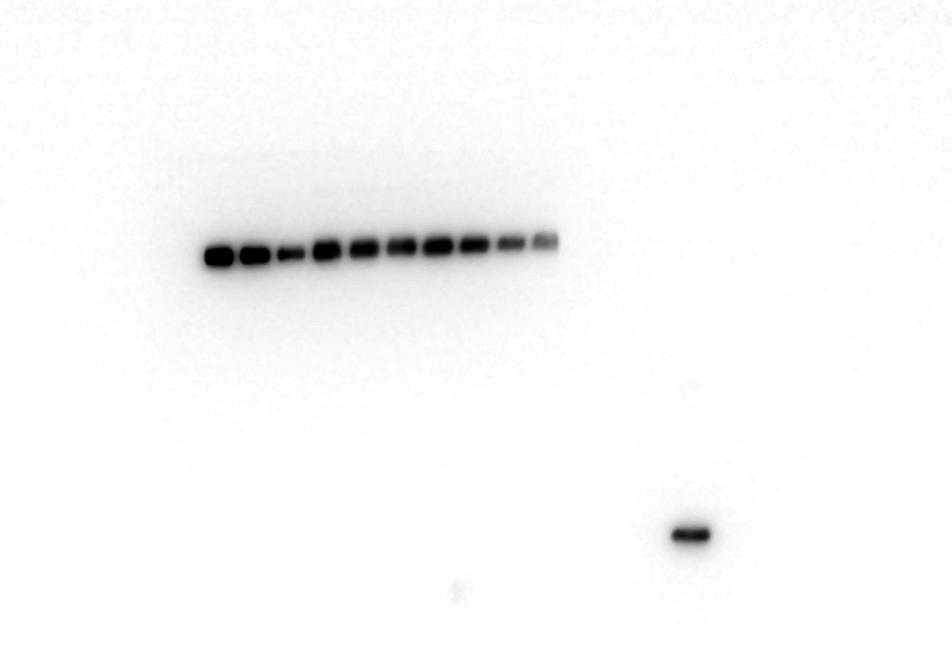

Supplement: Supplemental Information 5 [file peerj-11-15075-s005.zip › WB image/IGHA1_NExG.jpg]

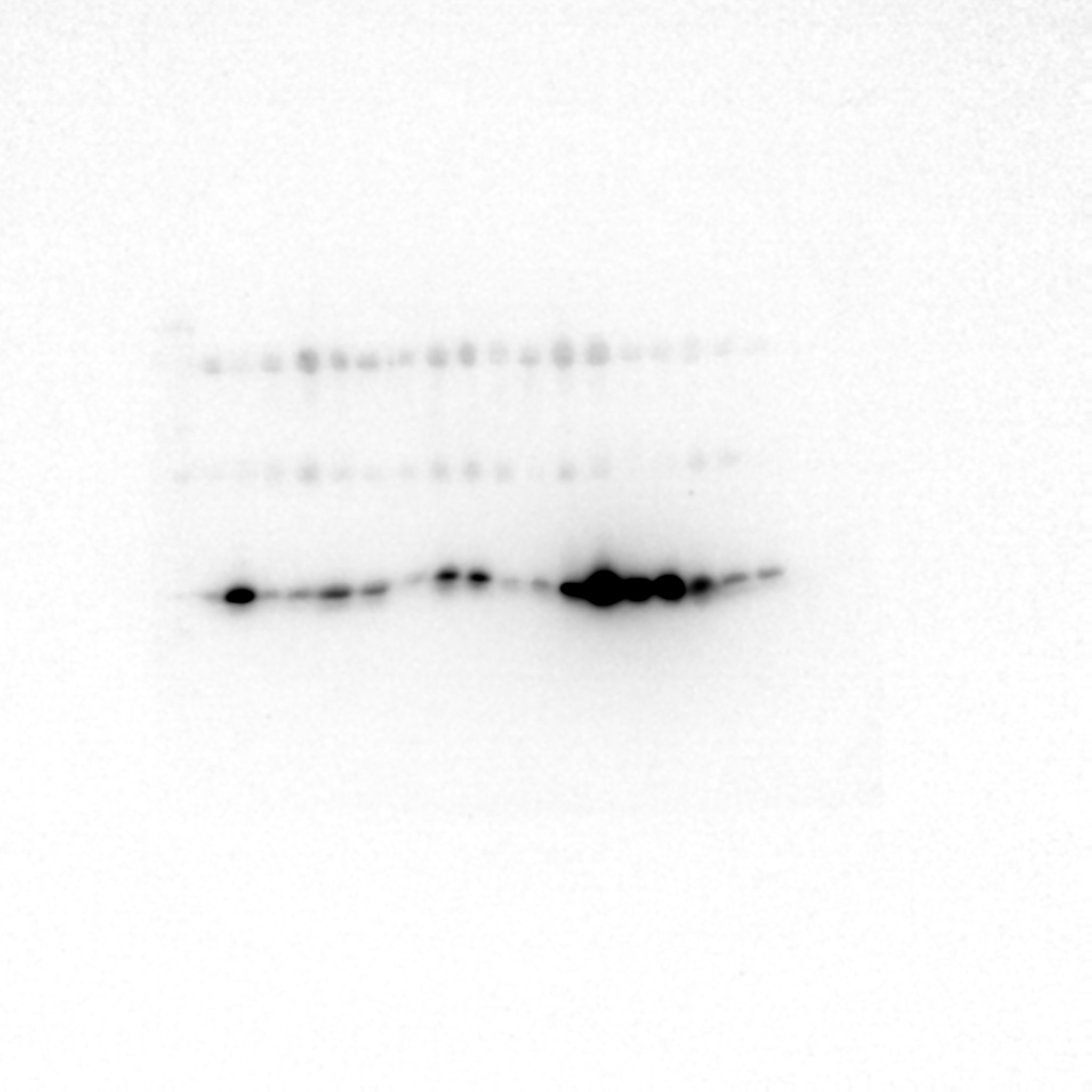

Supplement: Supplemental Information 5 [file peerj-11-15075-s005.zip › WB image/igk_itraq.jpg]

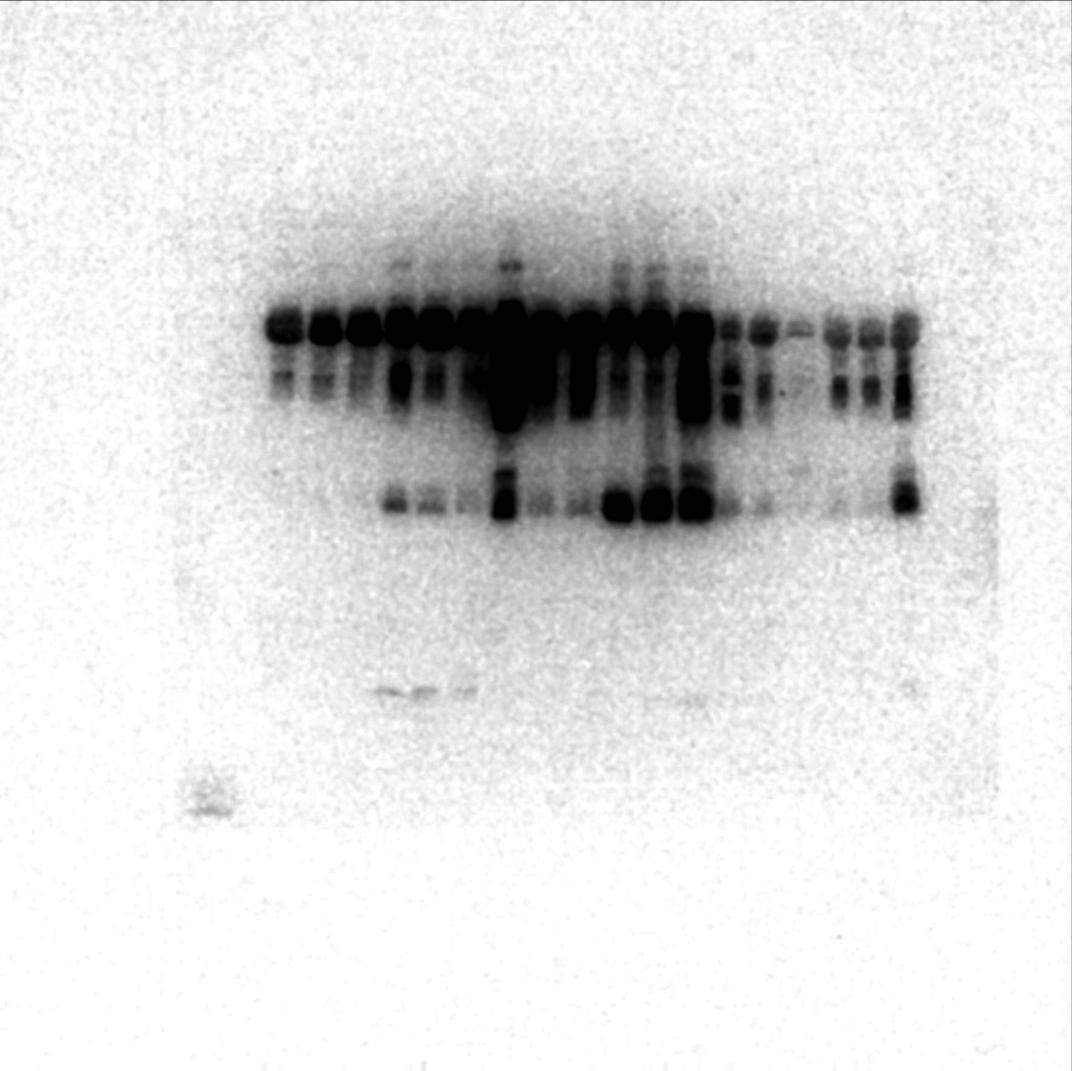

Supplement: Supplemental Information 5 [file peerj-11-15075-s005.zip › WB image/klk1_traq.jpg]

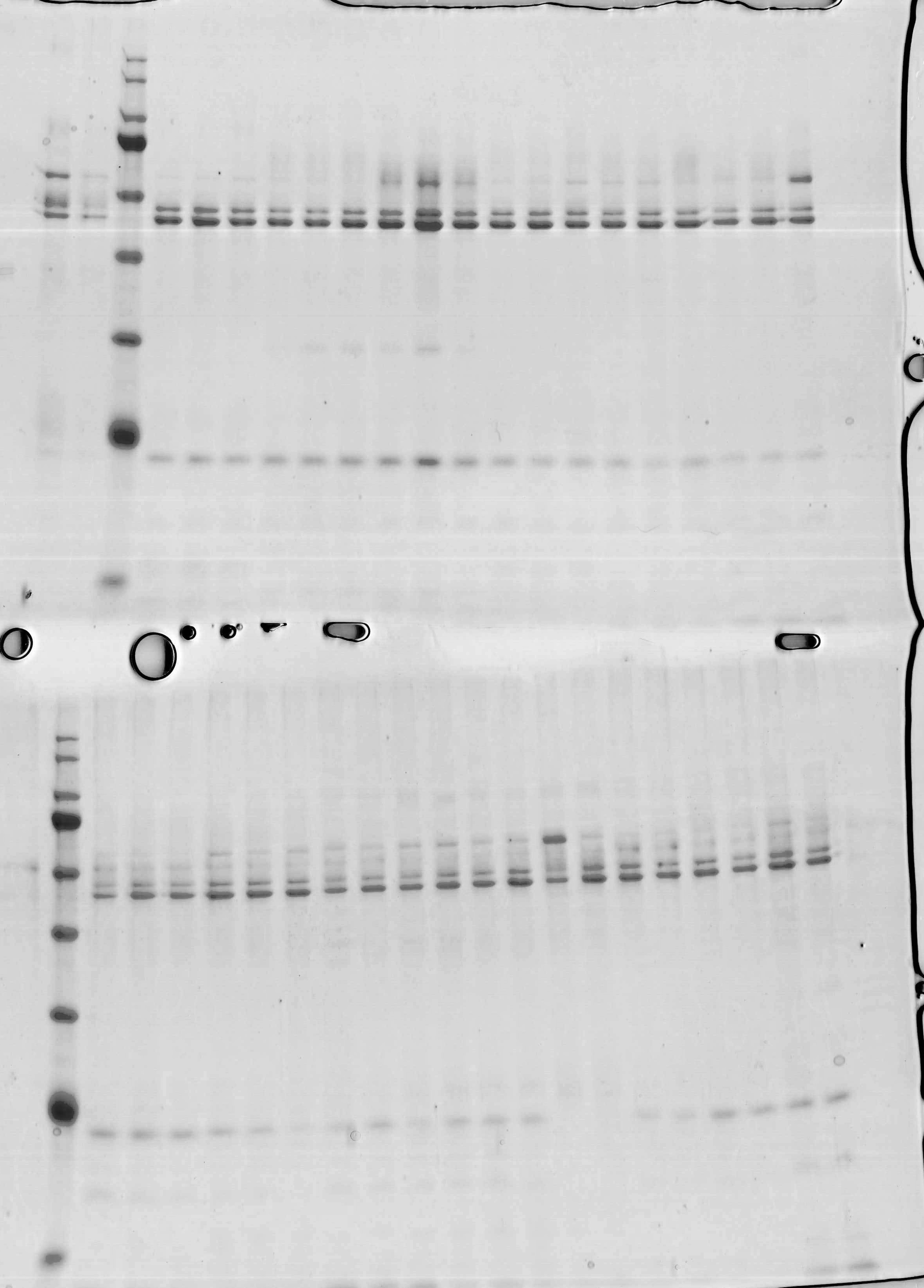

Supplement: Supplemental Information 5 [file peerj-11-15075-s005.zip › WB image/cbb gel.jpg]

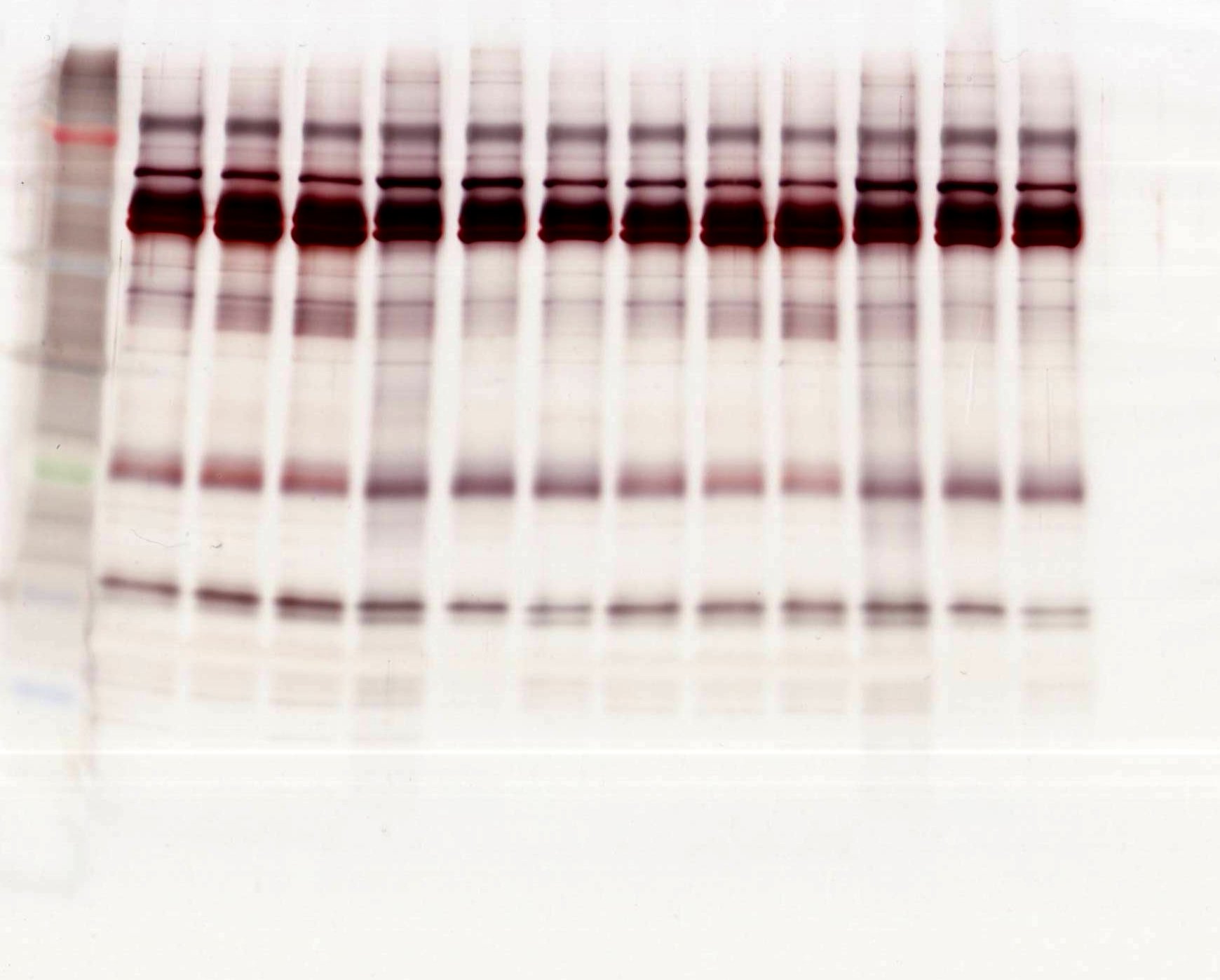

Supplement: Supplemental Information 5 [file peerj-11-15075-s005.zip › WB image/silver_stain.jpg]

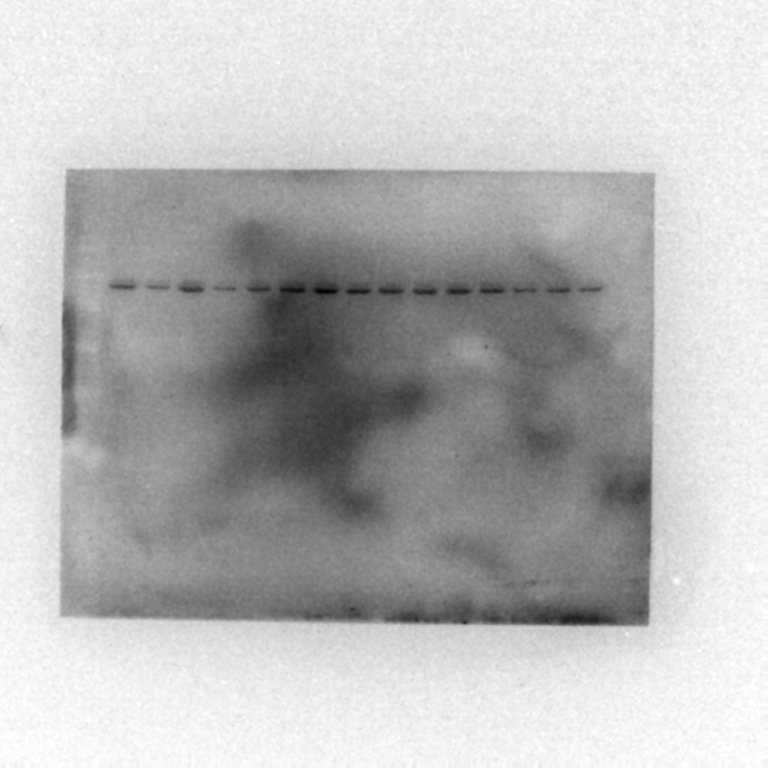

Supplement: Supplemental Information 5 [file peerj-11-15075-s005.zip › WB image/azgp1_itraq.jpg]
