# Supplementary material for: Analysis of IGHA1 and other salivary proteins post half marathon in female participants
Source: PeerJ. 2023 May 11;11:e15075. doi: 10.7717/peerj.15075 (PMC10183162; doi:10.7717/peerj.15075)
Supplement: Supplemental Information 5 [file peerj-11-15075-s005.zip › WB image/WB image info.pdf]

Fig2.C CBB stain gel

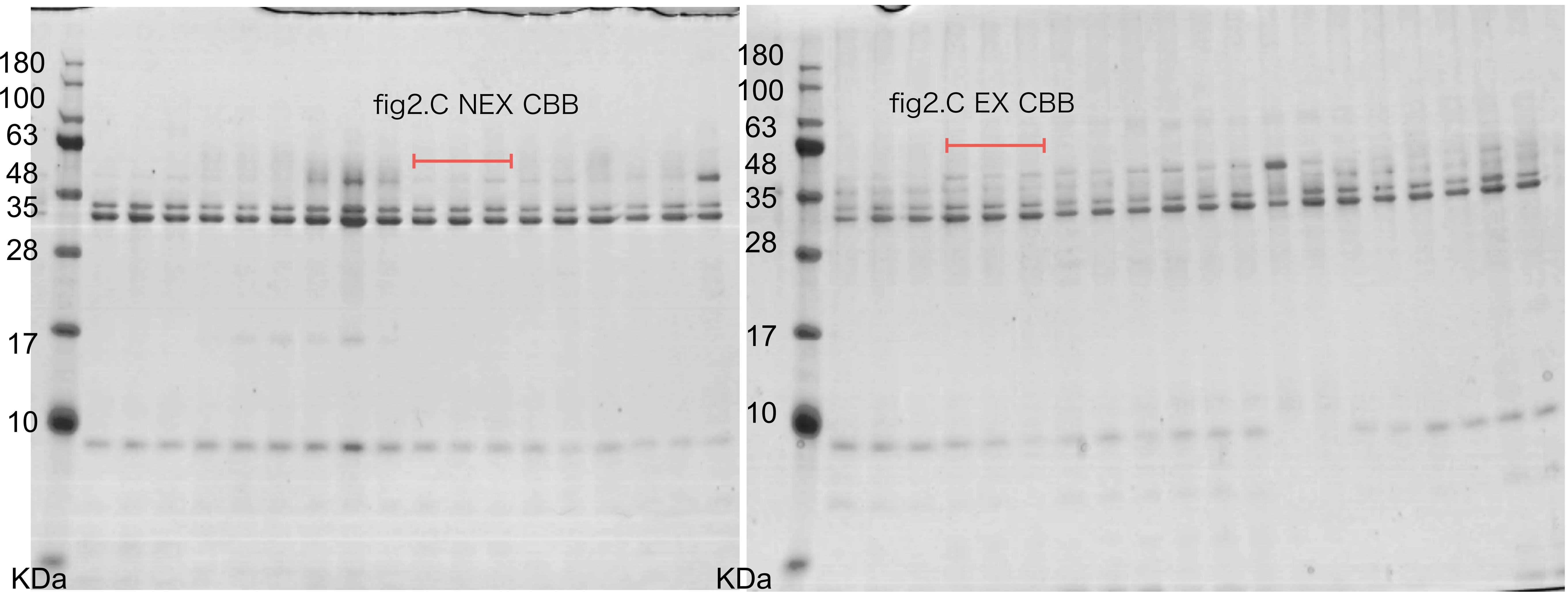

Raw data was saved in WB image folder named cbb gel

Fig2.C α IGHA1

IGHA1≒37KDa

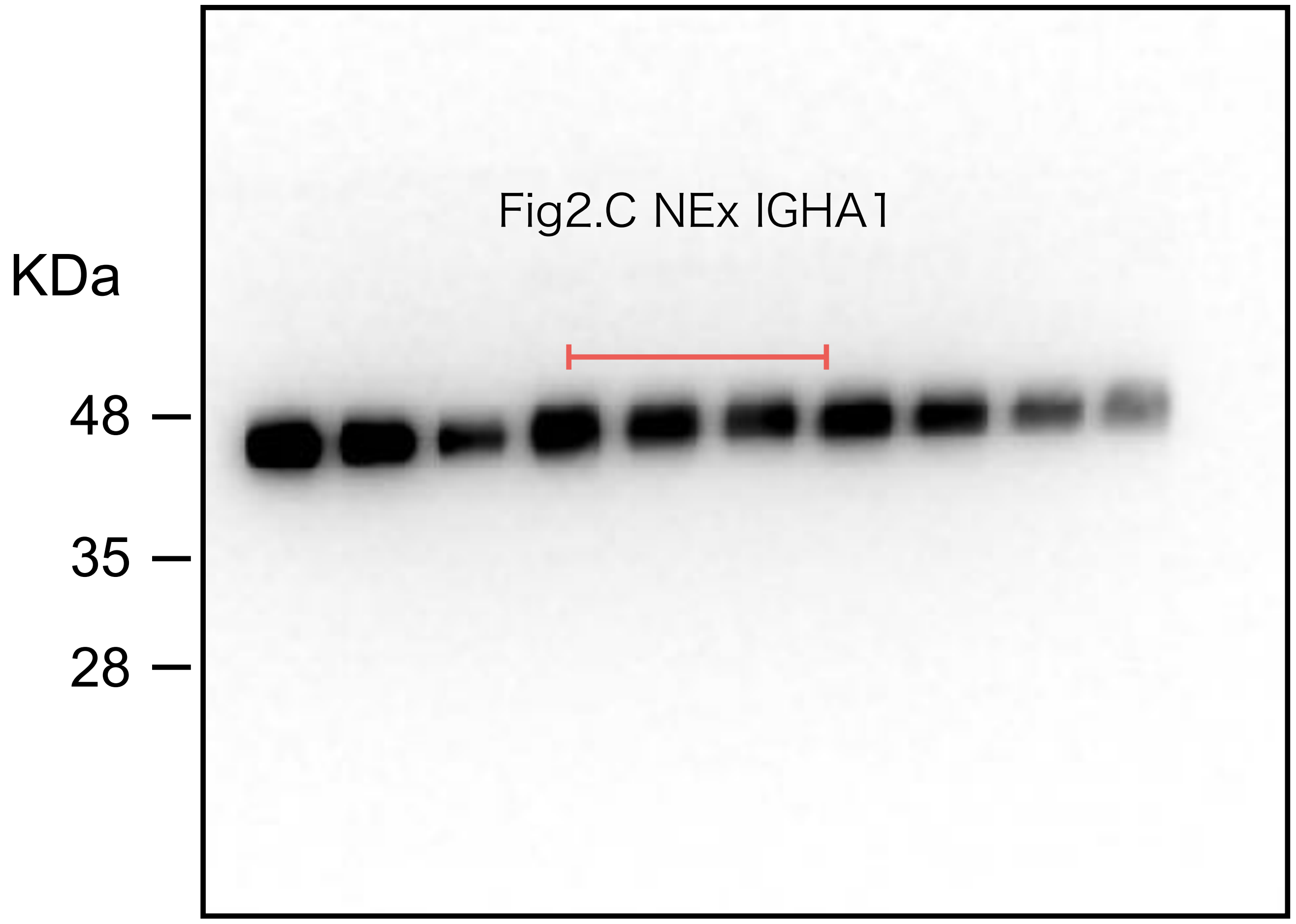

Raw data was saved in WB image folder named IGHA1_NExG

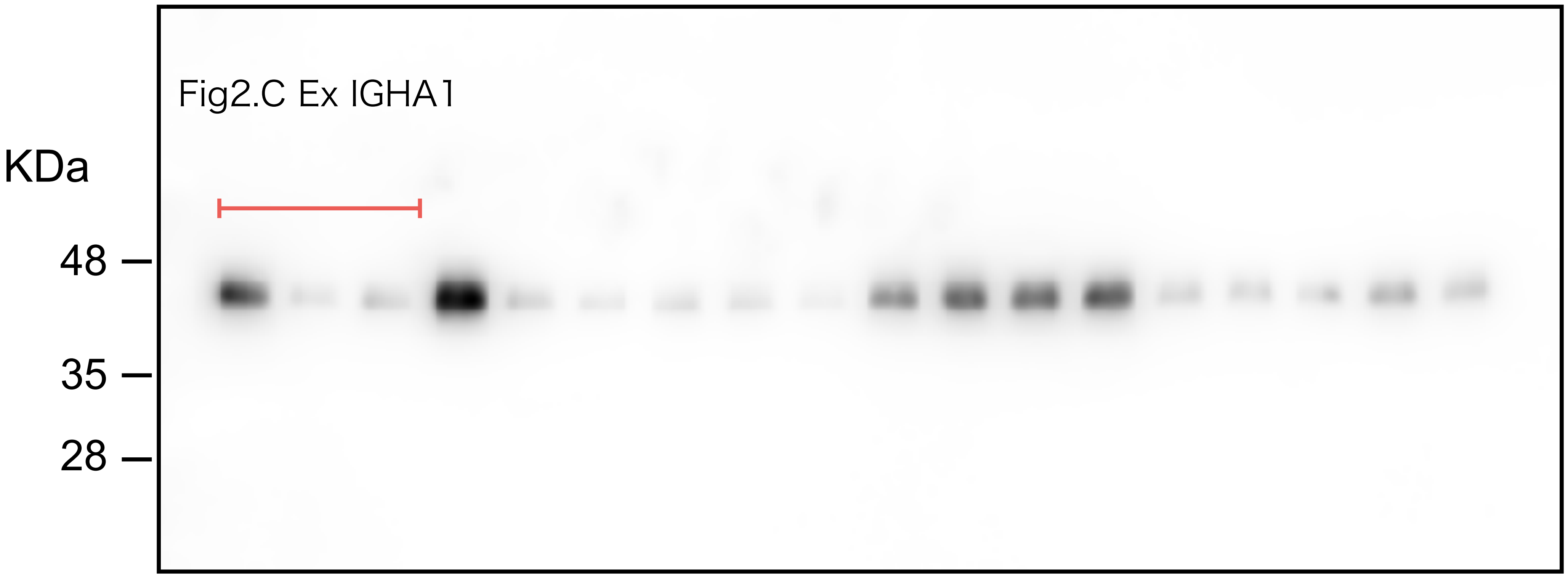

Raw data was saved in WB image folder named IGHA1_ExG

Fig3.D WB

KLK1≒28KDa

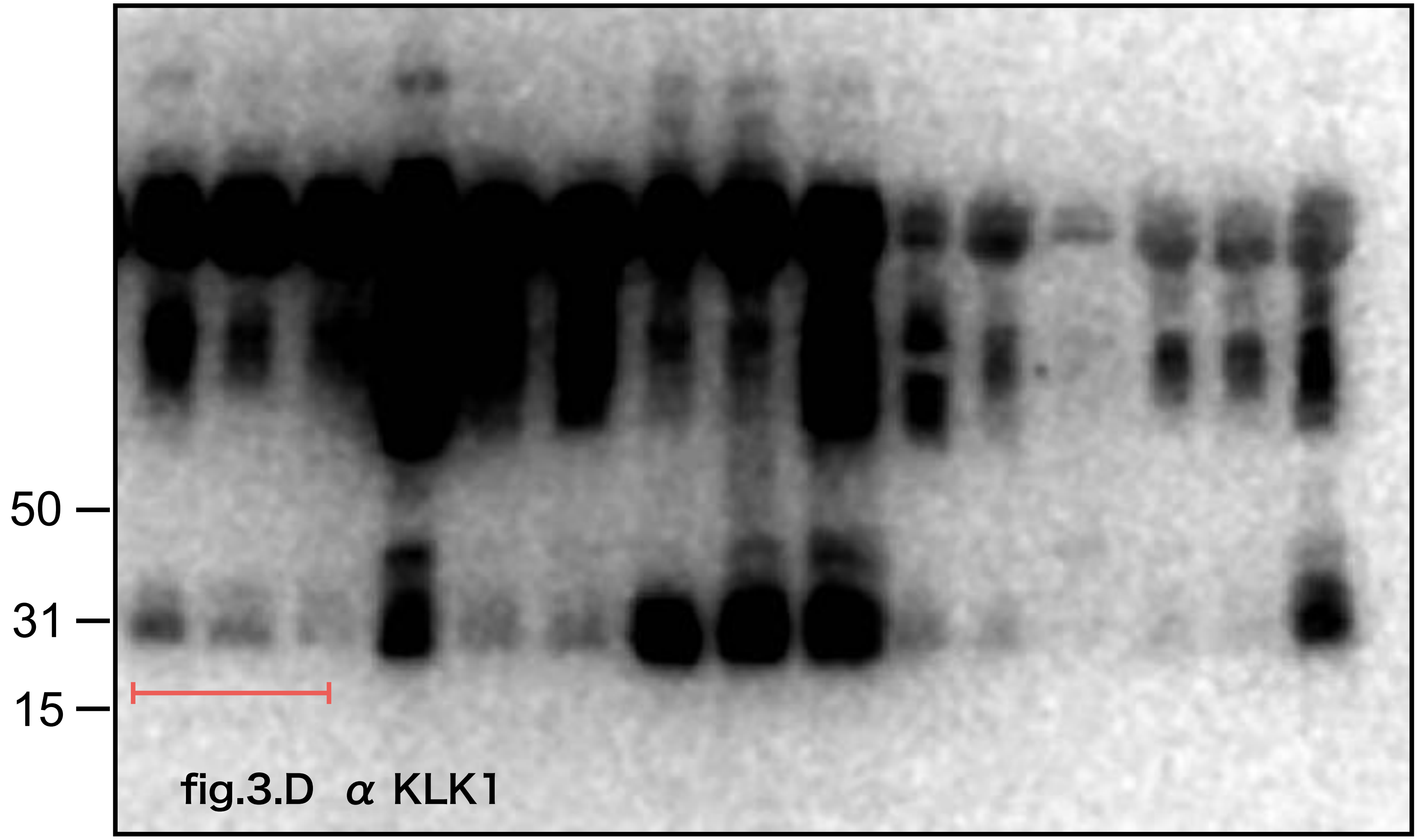

Raw data was saved in WB image folder named klk1_traq

AZGP1≒34KDa

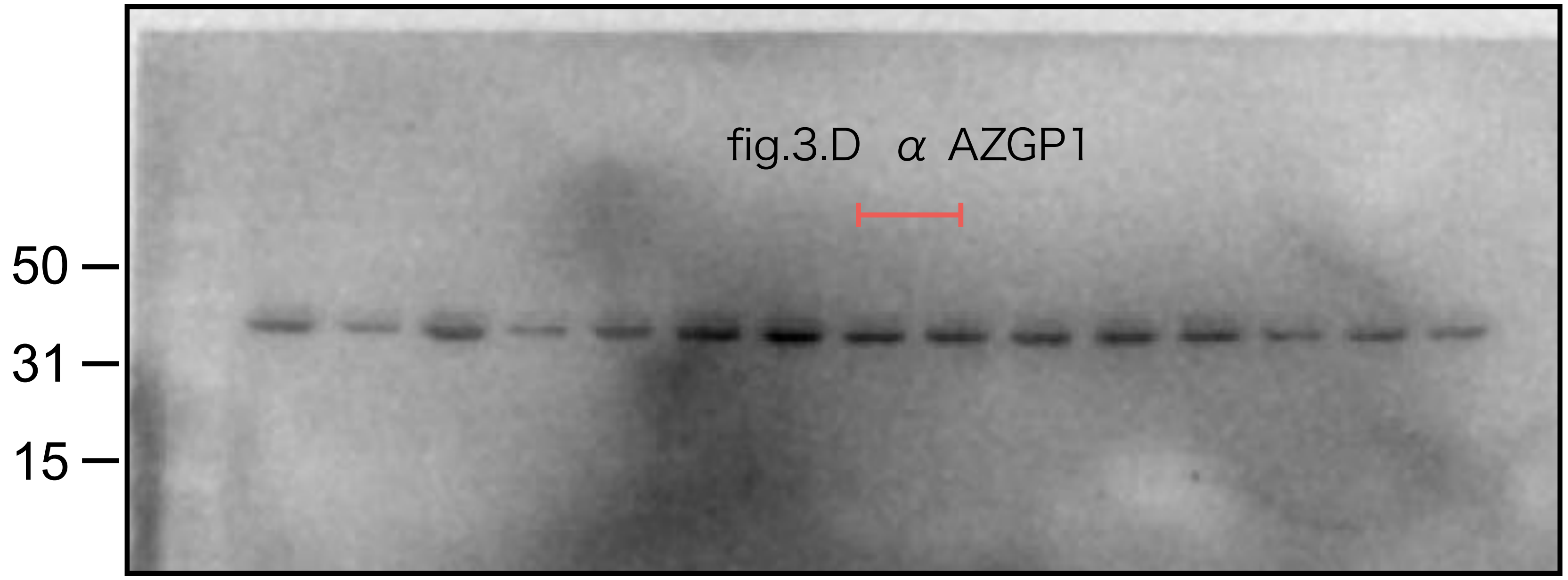

Raw data was saved in WB image folder named azgp1_itraq

Fig3.D WB

IGK≒23KDa

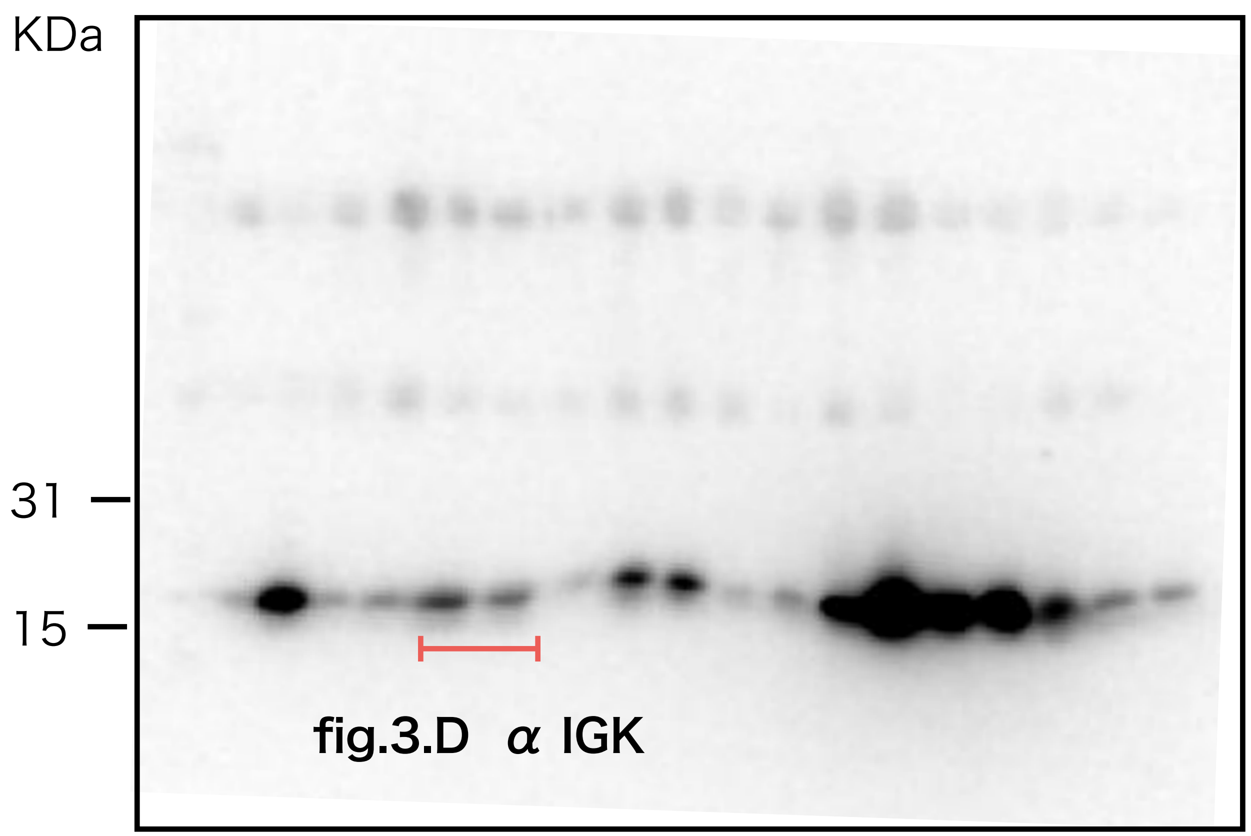

Raw data was saved in WB image folder named igk_itraq

CST4≒16KDa

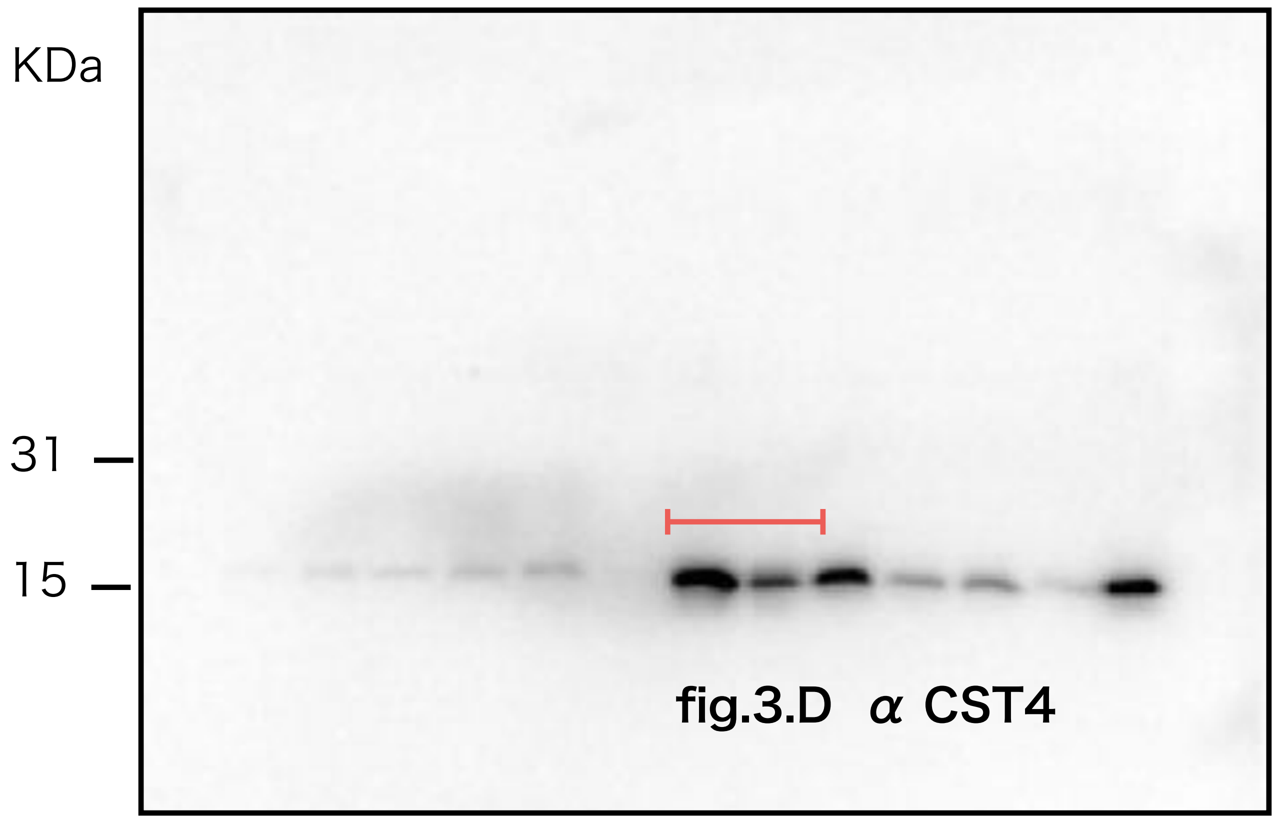

Raw data was saved in WB image folder named cst4_itraq

Figs1 silver satin

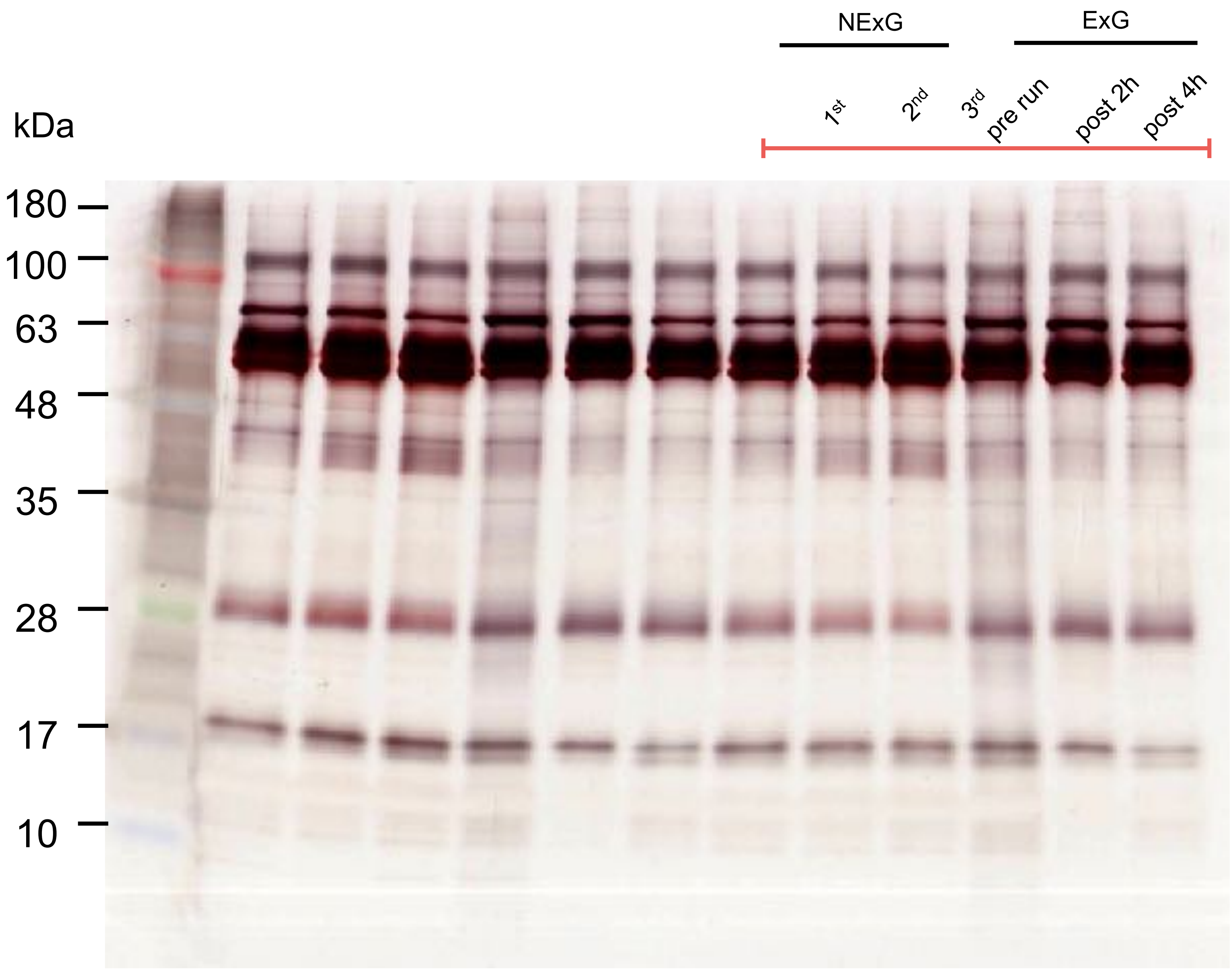

Raw data was saved in WB image folder named silver_stain
